# Acral vascular syndrome Lennert type T cell lymphoma—a case report

**DOI:** 10.1186/s43046-021-00063-7

**Published:** 2021-03-04

**Authors:** Kamal Bandhu Klanidhi, S. Ashwin Monian, Avinash Chakrawarty

**Affiliations:** Department of Geriatric Medicine, All India Institute of Medical Sciences (AIIMS), Room no. 3095A, New Delhi, 110029 India

**Keywords:** Acral vascular syndrome, Peripheral T cell lymphoma, Vitiligo

## Abstract

**Background:**

Acral vascular syndrome clinically presents as digital ischemia with Raynaud’s phenomenon and erythromelalgia but can be rarely seen in the malignant condition. Patients may present pain, permanent digital blanching or cyanosis, and desquamation or ulceration of the fingers. Acral vascular syndrome is rarely associated with lymphoid neoplasm and is associated with smoking, autoimmune connective tissue diseases, and vasculitis. Here, we describe a 79-year-old female who was diagnosed with vitiligo and peripheral T cell lymphoma Lennert type stage 4 with anemia of chronic disease with digital acral vascular syndrome.

**Case presentation:**

A 79-year-old female with vitiligo presented with gangrene of the distal extremities associated with pain and intermittent fever for 2 months. On evaluation, she was found to have anemia of chronic disease and generalized lymphadenopathy and diagnosed as peripheral T cell lymphoma Lennert type with bone marrow involvement and digital acral vascular syndrome.

**Conclusion:**

Acral vascular syndrome can be a presentation of lymphoma; if intervened earlier, the patient can be saved from the amputation of fingers or affected limb. Though it is a rare presentation of lymphoma, it should be considered if there is a rapid progression of gangrene. Early initiation of chemotherapy may result in the reduction of further progression of digital gangrene and thus prevents functional dependence on caregivers. In our patient, gangrene of other fingers was prevented even though it is an aggressive variant of T cell lymphoma.

## Background

Acral vascular syndrome due to malignancy is rarely reported and is usually seen in males. It can be presented with Raynaud’s phenomenon, acral cyanosis, and digital gangrene. Acral vascular syndrome is seen commonly with vasculitis and connective tissue disorder. Aggressive peripheral T cell lymphomas (PTCLs) have a poor prognosis with most histologies having a 5-year survival of 30% with chemotherapy. Early diagnosis and treatment can be associated with increased survival and reduced rate of amputation of digits. We describe a 79-year old female who was diagnosed with vitiligo and peripheral T cell lymphoma Lennert type stage 4 with anemia of chronic disease with digital acral vascular syndrome.

## Case presentation

**Fig. 1:**
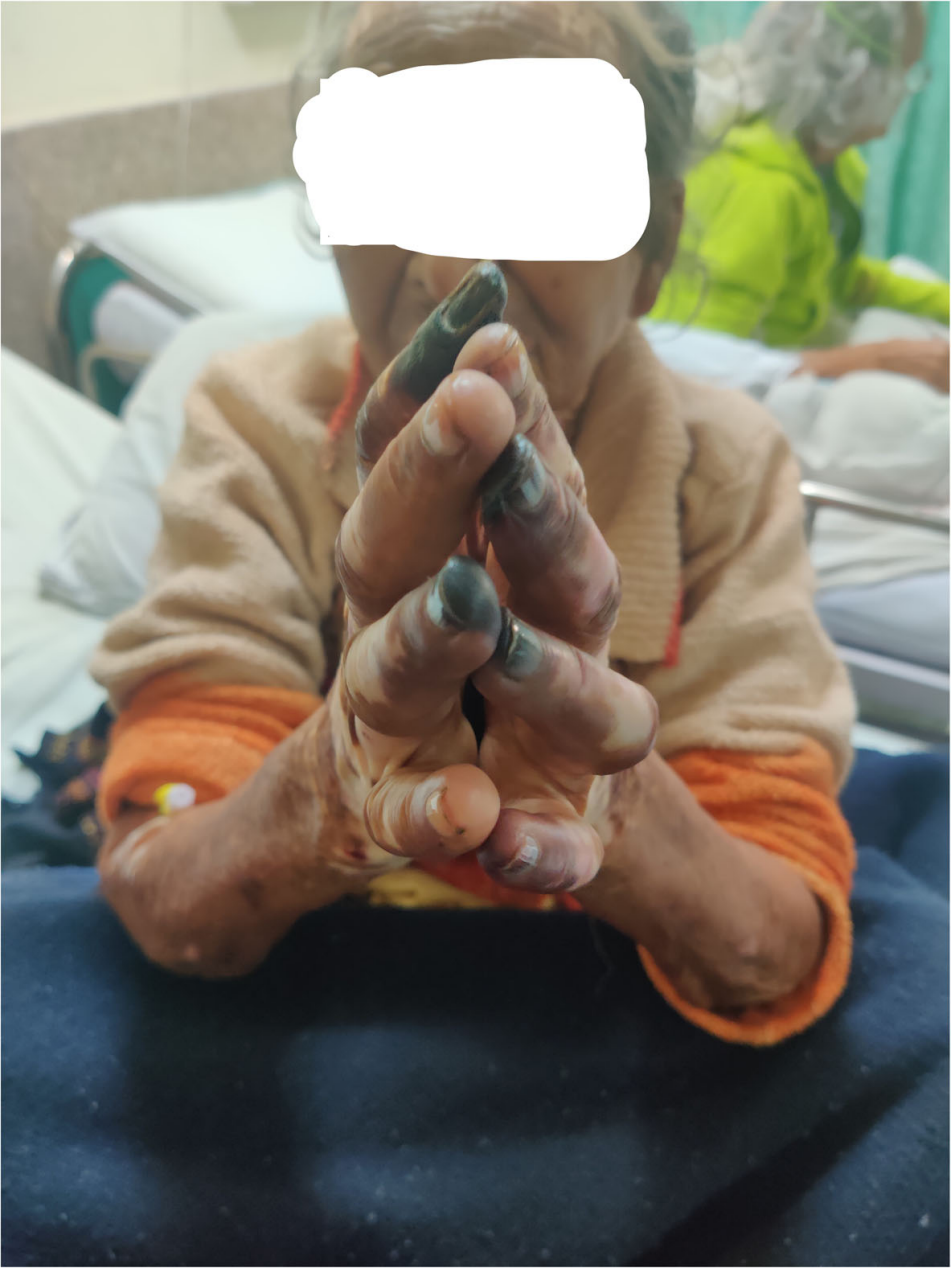
Clinical image showing gangrene in digits

A 79-year-old female having vitiligo for the last 40 years presented with complaints of a blackish discoloration of the right forearm and middle finger and necrotic areas over the shin associated with digital pain for 2 months (Fig. 1). She also complained of intermittent fever associated with chills for 2 months. She does not complain of joint pain, oral ulcer, skin rash, photosensitivity, grittiness of the eyes, and Raynaud’s phenomenon.

On examination, pallor, multiple irregular ulcers with blackish eschar and crusting over the bilateral shin, claves, and dorsum of the right hand were present. Dry gangrene of the right index and middle finger with demarcation line was found with vitiligo. Oral examination shows atrophy of the papillae and angular cheilitis. On routine investigation, hemoglobin of 9 g/dl, total leucocyte count of 15,000 with neutrophilic predominance and increased alkaline phosphatase, raised procalcitonin, and hyponatremia rest another parameter are normal. Further, evaluation of peripheral smear shows normocytic to microcytic with mild anisocytosis, ferritin 1742, vitamin B12 > 128, folate 10.1, lactate dehydrogenase 483, normal thyroid hormone level, negative for HIV1 and 2, anti-HCV Ab, and HBsAg.

Bilateral upper limb and lower limb arterial Doppler were done which shows triphasic flow suggestive of normal study. 2D ECHO was done which shows normal valve and normal left ventricular systolic function with an ejection fraction of 60%

18F-FDG whole-body PET-CT was done which reveals hypermetabolic cervical, axillary, mediastinal, and abdominopelvic lymph nodes and diffuse splenic and marrow metabolic activity suggestive of lymphoma or tuberculosis.

**Fig. 2:**
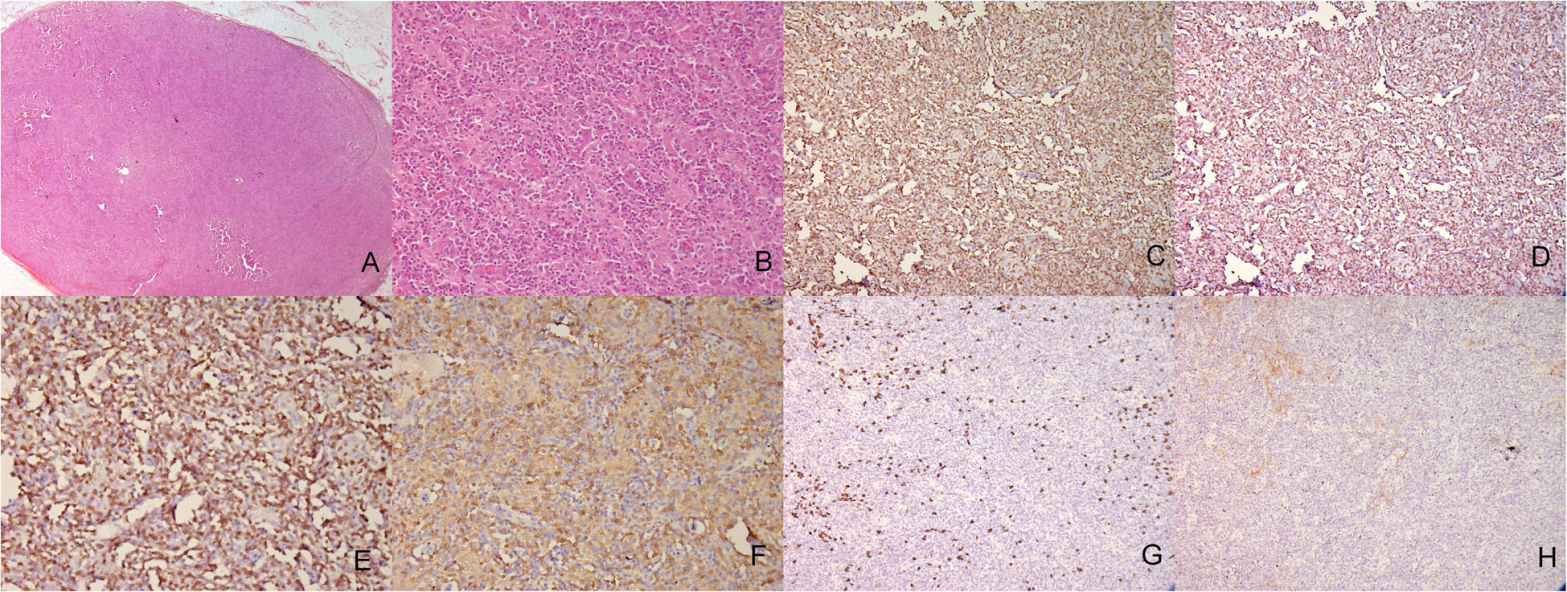
Lymph node biopsy shows effacement

Lymph node biopsy shows effacement of the lymph nodal architecture by a polymorphous population of cells comprising histiocytes, eosinophils, and atypical intermediate-size lymphoid cells (Fig. 2). The histiocytes are ill-defined granuloma. Atypical cells are positive for CD3, CD8 (>CD4), and CD7 while negative for CD20. There is a partial loss of CD5 with few cells positive for CD4. K67 proliferation index is approximately 40–50%. The overall features are suggestive of peripheral T cell lymphoma Lennert type.

**Fig. 3:**
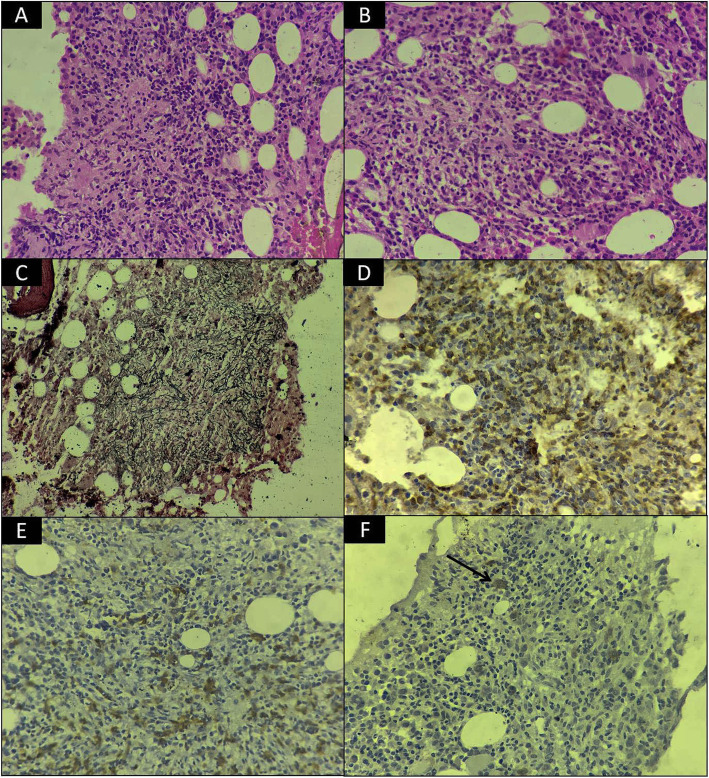
Showing bone marrow biopsy smear

Bone marrow aspirate smear shows myeloid preponderance with a M:E ratio of 4:1 (Fig. 3). Bone marrow biopsy shows 2 to 3 ill-formed epithelial granulomas with multiple lymphoid aggregates. Reticulin stain was suggestive of focal grade II fibrosis. CD3 shows small lymphoid aggregates in addition to interstitial cells. CD30 is positive only in occasional cells. CD20 shows interstitial B cells, suggestive of bone marrow involvement in the case of peripheral T cell lymphoma.

The final diagnoses are vitiligo and peripheral T cell lymphoma Lennert type stage 4 with anemia of chronic disease with digital acral vascular syndrome.

The patient was started on etoposide, procarbazine, cyclophosphamide, and prednisone, and digital gangrene has become stagnant and was discharged to follow up for further cycles of chemotherapy.

## Conclusion

Acral vascular syndrome clinically presents as digital ischemia with Raynaud’s phenomenon and erythromelalgia but can be rarely seen in the malignant condition. The patient may present pain, permanent digital blanching or cyanosis, and desquamation or ulceration of the fingers. Acral vascular syndrome is seen to be associated with smoking, autoimmune connective tissue diseases, vasculitis, and local injuries such as hammer syndrome. It is rarely associated with lymphoid neoplasm [1]. Peripheral T cell lymphomas (PTCLs) arise from post-thymic lymphocytes and have varied clinical presentations [2].

Age-adjusted incidence rates are higher in males than in females [3]. PTCL-not otherwise specified (PTCL-nos) is a highly heterogeneous group of nodal lymphomas which can involve extranodal sites like the skin and gastrointestinal tract (GIT).

Lennert lymphoma (LL) is a lymphoepithelioid morphological variant of peripheral T cell lymphoma—not otherwise specified (PTCL/NOS) [4]. In the fourth edition of the WHO classification, LL was finally classified as a PTCL/NOS morphological variant characterized by clear cell cytology, cytotoxic phenotype, and abundant, when not prominent, epithelioid cells reaction [5].

Aggressive PTCLs have a poor prognosis with most histologies having a 5-year survival of 30% with chemotherapy. High-dose chemotherapy and ASCT have been used as a consolidation strategy in eligible patients with a chemo-sensitive disease. Following regimen is used for the treatment of PTCL include CHOEP (Cytoxan, Adriamycin, vincristine, etoposide, prednisone), CHOP (Cytoxan, Adriamycin, vincristine, prednisone), COEP (Cytoxan, vincristine, etoposide, prednisone), and PEGS (cisplatin, etoposide, gemcitabine, methylprednisolone). The Food and Drug Administration (FDA) approved few single agents for the treatment of relapsed refractory PTCL which include pralatrexate, romidepsin, belinostat, brentuximab vedotin, mogamulizumab, and chidamide. Non-FDA–approved agents for the treatment of relapsed PTCL-nos include brentuximab vedotin, bendamsutine, alemtuzumab, and lenalidomide.

Specific targeted therapies include CD30, CD25, CCR4, CD38, directed therapies, JAK/STAT inhibitors, PI3Kinase inhibitors, immunotherapies, and immune checkpoint inhibitors [6].

Treatment initiation at an early stage can prevent gangrene of other fingers. In our patient, gangrene of other fingers was prevented even though it is an aggressive variant of T cell lymphoma.

## Data Availability

Not applicable.

## Abbreviation

PTCL: Peripheral T cell lymphoma
NOS: Not otherwise specified
HIV: Human immunodeficiency virus
HCV: Hepatitis C virus
Ab: Antibody
HBsAg: Hepatitis B virus surface antigen
ECHO: Echocardiography
FDG: Fluro deoxy glucose
PET CT: Positron emission tomography
ASCT: Autologous stem cell transplantation
M:E: Myeloid:erythroid
